# An approach for exploring interaction between two proteins *in vivo*

**DOI:** 10.3389/fphys.2014.00162

**Published:** 2014-04-29

**Authors:** Hiroshi Qadota, Guy M. Benian

**Affiliations:** Department of Pathology, Emory UniversityAtlanta, GA, USA

**Keywords:** protein-protein interactions, yeast 2-hybrid, *C. elegans*, transgenics, kindlin, ILK, protein localization

## Abstract

We describe a strategy for exploring the function of protein-protein interactions in striated muscle *in vivo*. We describe our experience using this strategy to study the interaction of UNC-112 (kindlin) with PAT-4 (integrin linked kinase). Random mutagenesis is used to generate a collection of mutants that are screened for lack of binding or gain of binding using a yeast 2-hybrid assay. The mutant proteins are then expressed in transgenic *C. elegans* to determine their ability to localize in the sarcomere. We emphasize two advantages of this strategy: (1) for studying the interaction of protein A with protein B, when protein A can interact with multiple proteins, and (2) it explores the function of an interaction rather than the absence of, or reduced level of, a protein as can be obtained with null mutants or knockdown by RNAi. We propose that this method can be generalized for studying the meaning of a protein-protein interaction in muscle for any system in which transgenic animals can be generated and their muscles can be imaged.

## Background—UNC-112 (kindlin) and PAT-4 (ILK)

The model genetic system *C. elegans* is an excellent platform for learning new information about striated muscle, including new components of the sarcomere, requirements for assembly and maintenance of the sarcomere, and mechanisms of the regulation of the contraction/relaxation cycle (Waterston, 1988; Moerman and Fire, 1997; Moerman and Williams, 2006; Qadota and Benian, 2010). Because of the evolutionary conservation of sarcomere structure and components, these studies in *C. elegans* have relevance to our understanding of the inherited cardiomyopathies, most of which are due to mutations in sarcomere proteins (Benian and Epstein, 2011; Epstein and Benian, 2012). In *C. elegans* the myofibrils are organized such that all thick filament organizing centers (M-lines) and thin filament attachment sites (dense bodies analogous to Z-disks) are anchored to the muscle cell membrane. These integrin-based muscle adhesion sites are ideal models for costameres of vertebrate striated muscle and focal adhesions of non-muscle cells. Many components of *C. elegans* myofibrils and their attachment structures have been defined. Most were first identified through mutation—most falling into one of two phenotypic classes. In one class, the uncoordinated or “Unc” class, due to mutation in any one of ~40 genes, animals develop into adults but they are slow moving or paralyzed (Waterston et al., 1980; Zengel and Epstein, 1980). The second class, called “Pats” (paralyzed arrested at two-fold) result from mutation in one of 20 genes; embryos do not move in the eggshell and elongation arrests at the two-fold embryonic stage (Williams and Waterston, 1994; Meissner et al., 2009).

Our laboratory has helped discover an interacting network of proteins that extend from integrin in the muscle cell membrane inward toward myosin at M-lines, and actin at dense bodies (Qadota and Benian, 2010; Warner et al., 2011, 2013; Lecroisey et al., 2013). One component of the attachment structures is UNC-112 (kindlins in mammals). UNC-112 is part of a conserved four-protein complex that associates with the cytoplasmic tail of integrin. Although a kindlin was first discovered in humans in 1994 (Wick et al., 1994), it was not until the description of UNC-112 in 2000 (Rogalski et al., 2000) that we learned that kindlins have a role in integrin adhesion complexes. In contrast to *C. elegans*, which has one kindlin (UNC-112), humans have three kindlins, each encoded by a separate gene (Meves et al., 2009). Inherited mutations in kindlin-1 result in a serious skin disease (Kindler syndrome) (Siegel et al., 2003), and mutations in kindlin-3 result in severe dysfunction of both platelets and leukocytes (leukocyte adhesion deficiency type III) (Moser et al., 2009; Svensson et al., 2009). Kindlin-2 is expressed in the heart and localized to intercalated disks and costameres. Loss of function of kindlin-2 in zebrafish results in severe abnormalities of heart development and function (Dowling et al., 2008).

**Figure 1:**
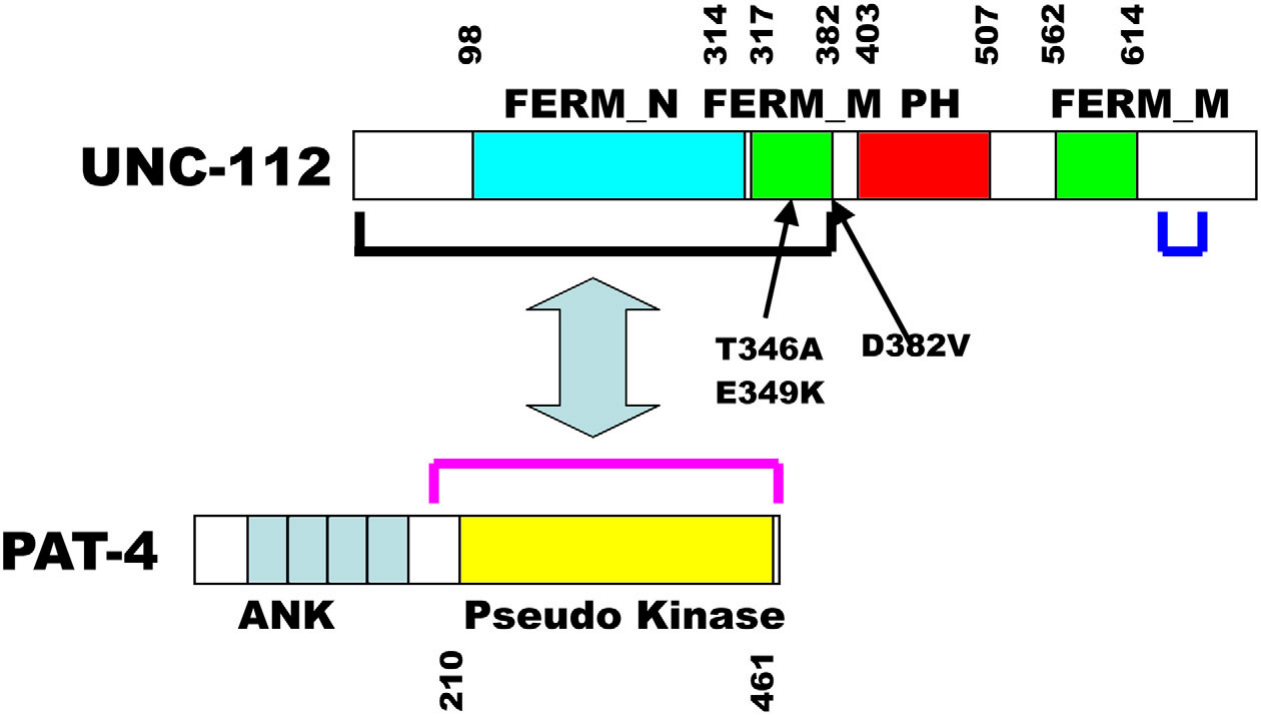
**Schematic representation of UNC-112 indicating predicted domains, mutation sites, and interaction with PAT-4**. Domains of each protein, indicated by colored rectangles were predicted by PFAM, with boundaries indicated by residue numbers. The black and red brackets indicate the minimal regions that are required for interaction of UNC-112 with PAT-4, respectively. In UNC-112, the D382V mutation prevents UNC-112 from interacting with PAT-4; the T346A and the E349K mutations each prevent interaction of the N-terminal half of UNC-112 with the C-terminal half of UNC-112. The blue bracket denotes the 30 residue region (633–663) containing 5 residues that when mutated singly permit the N-terminal half of UNC-112 containing T346A to interact with the C-terminal half of UNC-112.

**Figure 2:**
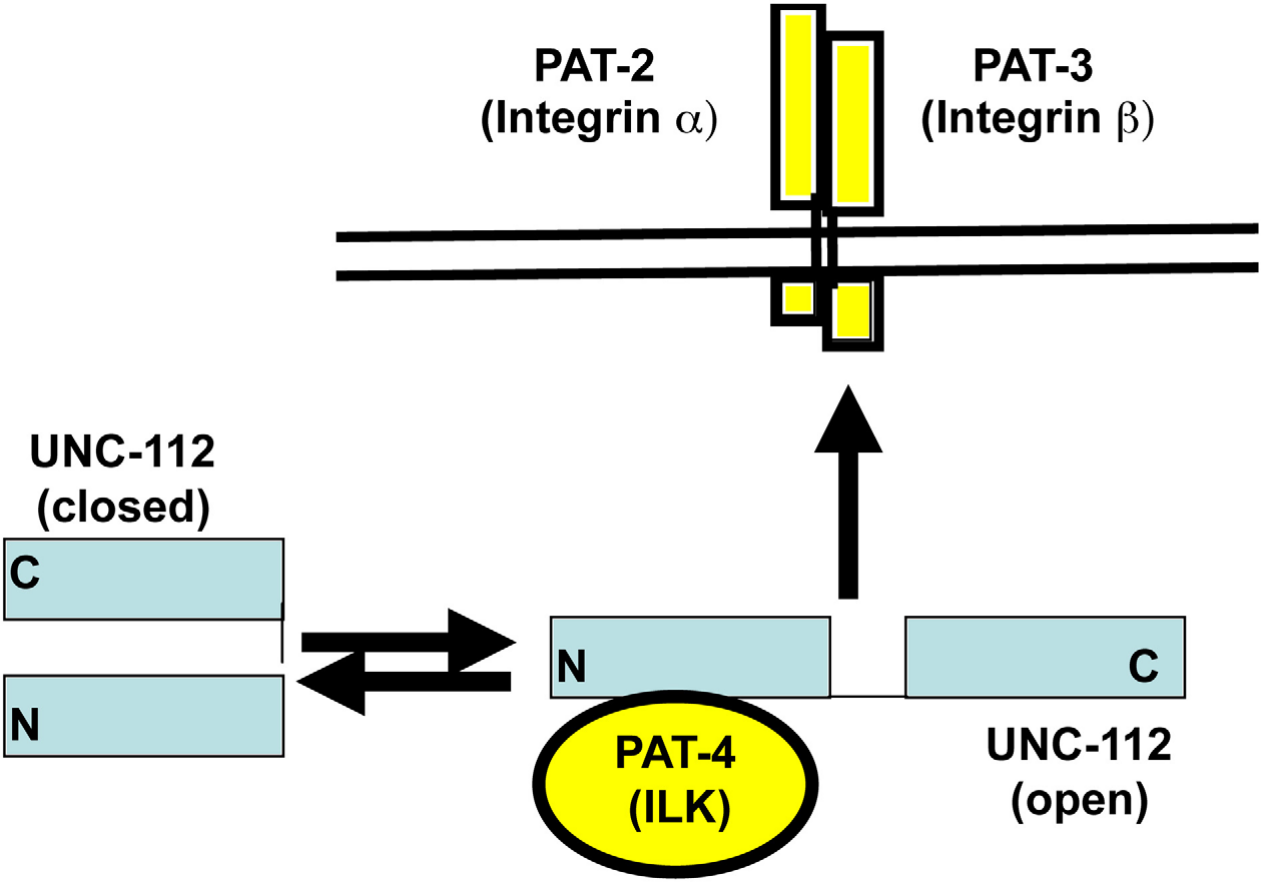
**Conformation-based model for the interaction of UNC-112 with integrin**. Based on yeast 2-hybrid, biochemical and mutational analysis, it is hypothesized that UNC-112 exists in two conformations, closed and open, and that only the open form can interact with the cytoplasmic tail of integrin at the base of adhesion sites in muscle. Further, the conversion to the open active form is promoted by binding of UNC-112 to PAT-4. (modified from Qadota et al., 2012).

In embryonic muscle, proper localization of UNC-112 to integrin adhesion sites requires PAT-4 (the nematode ILK ortholog) (Rogalski et al., 2000); the converse is also true: the proper localization of PAT-4, requires UNC-112 (Mackinnon et al., 2002). We sought to understand the molecular basis of this requirement. We used the cytoplasmic tail of PAT-3 (β-integrin) as bait to screen a yeast 2-hybrid library of *C. elegans* cDNAs, and recovered as the only interacting protein, UNC-112 (Qadota et al., 2012). This interaction was confirmed in two ways: (1) glutathione agarose beads coated with GST-PAT-3, but not GST, were able to pull out UNC-112 from a worm lysate; and (2) similar beads were able to bind to purified bacterially expressed MBP-UNC-112-His. When the C-terminal half of UNC-112 was used to screen the 2-hybrid library, all positive clones were cDNAs encoding UNC-112. Additional 2-hybrid and *in vitro* binding data indicate that the N-terminal and C-terminal halves of UNC-112 interact with each other. By either 2-hybrid or *in vitro* binding assays, PAT-4 does not interact with the cytoplasmic tail of PAT-3. PAT-4 was shown to interact with the N-terminal half of UNC-112 (Mackinnon et al., 2002) (Figure 1). All these data led us to hypothesize that UNC-112 exists in closed inactive, and open active conformations; that binding of PAT-4 to the UNC-112 N-terminal half results in an opening up of UNC-112, and consequent binding of the UNC-112/PAT-4 complex to the cytoplasmic tail of PAT-3 (Qadota et al., 2012) (Figure 2). In support of this model, we could demonstrate competition between the UNC-112 C-terminal half and PAT-4 for binding to the UNC-112 N-terminal half (Qadota et al., 2012).

Our model that UNC-112 engages in intramolecular interaction to form a closed conformation is compatible with the phenomenon of intramolecular masking of other ERM proteins such as ezrin, radixin and moesin, which like UNC-112 contain a FERM domain. In the case of ERM proteins, the FERM domain is unable to interact with membrane proteins when it is bound to its ~100 residue C-terminal tail (Reczek and Bretscher, 1998). It should also be pointed out that the complete structure of any kindlin family protein has not been reported. We only know the structures of two small portions of kindlins; there is an NMR structure of the ~100 residue N-terminus (or Fo domain) of mouse kindlin-1 (Goult et al., 2009), an NMR structure (Liu et al., 2011) and a preliminary crystal structure (Lee et al., 2011) of the 100 residue PH domain human kindlin-2, and a crystal structure of the PH domain of mouse kindlin-1 (Yates et al., 2012b). Recently, SAXS analysis of mouse kindlin-3 has been reported (Yates et al., 2012a). The proposed model suggests that the two halves of FERM-M (aka F2 and F3) that are split by the PH domain, are physically next to each other. Since these two halves are in the N- and C-terminal halves of these molecules, the SAXS data is compatible with our “closed conformation” model.

## Evaluation of the meaning of protein-protein interaction *in vivo*

If our model was correct, we reasoned that an UNC-112 mutant that cannot bind to PAT-4, would not localize to muscle adhesion sites *in vivo*. We screened for an UNC-112 mutant protein that cannot bind to PAT-4 by yeast 2-hybrid assay, and then tested for its localization by generating and analyzing transgenic worms. Error prone PCR (Cadwell and Joyce, 1992) was used to introduce mutations randomly into the N-terminal half of UNC-112, the portion of UNC-112 known to interact with PAT-4. In order for PCR to introduce more than the usual errors, the fidelity of Taq DNA polymerase is decreased by modifying the reaction mixture to contain Mn2+ (0.5 mM) and increasing the concentration of Mg2+ (e.g., to 7.5 mM). Varying the amount of template DNA, empirically, controls the extent of mutagenesis. The concentration of template is inversely proportional to the level of mutations; a low level of template results in more mutations.

The PCR primers had 30 nt of sequence that overlapped a linearized 2-hybrid prey plasmid containing the wild type C-terminal half of UNC-112. A mixture of the PCR fragment and linearized prey, was transformed into yeast harboring a bait plasmid containing the cytoplasmic tail of PAT-3. Generation of full-length UNC-112 prey clones occurred via recombination in the yeast cells, due to the 30 bp of overlapping sequence (Takita et al., 1997). Transformed yeast cells were placed onto selective media, and those that grew indicated interaction between mutagenized UNC-112 and PAT-3. This step was crucial for eliminating clones with premature stop mutations or other trivial reasons. From 86 colonies, the PAT-3 bait was eliminated, and the UNC-112 prey plasmids were isolated. These UNC-112 prey clones were transformed separately into yeast strains carrying either the PAT-3 bait, or the PAT-4 bait. From the 86 UNC-112 preys, 32 preys showed interaction with PAT-3, and one showed no interaction with PAT-4. This clone had two mutations—R73L and D382V. Prey clones of UNC-112 harboring each of these single amino acid changes were generated and tested for PAT-4 binding: only D382V eliminated binding to PAT-4 (Figure 1). The N-terminal half of UNC-112 containing D382V continued to interact with a wild type version of the C-temrinal half of UNC-112.

**Figure 3:**
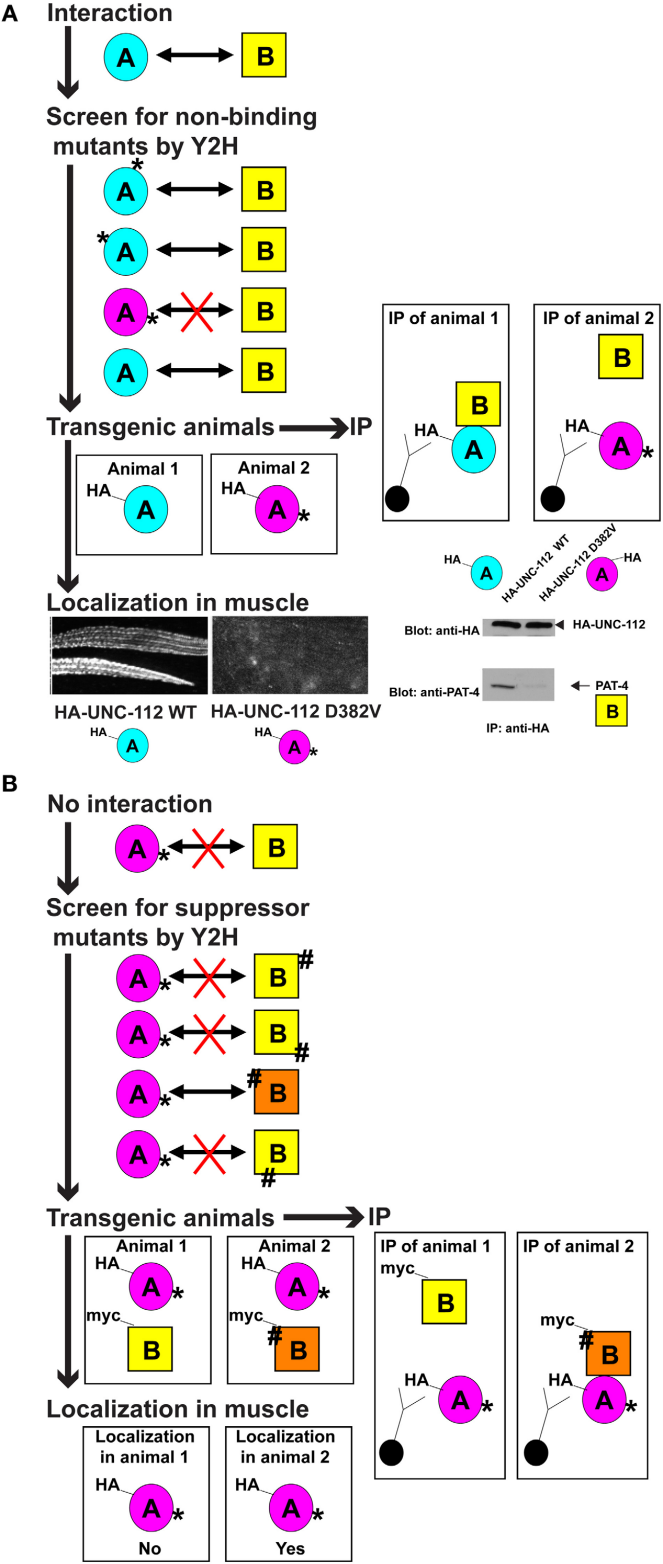
**Generalized description of the method. (A)** Isolation and characterization of a mutant in protein A that does not bind to protein B. From top to bottom: protein A is known to interact with protein B. Random mutagenesis is used to generate a collection of protein A mutants (mutations indicated by ^*^ at various positions in the protein), and these are tested by yeast 2-hybrid assays for interaction with protein B. One or more such protein A mutants (^*^ and purple) is found to not interact with protein B (denoted by a red X). Transgenic animals are generated that express either wild type protein A (blue), or mutant protein A (purple), each with an immunotag (e.g., HA, as shown). These transgenic animals are used in two types of experiments. As indicated below, using an antibody that detects the immunotag, the requirement of the interaction of protein A with protein B for localization of protein A, is assessed by immunofluorescent detection in muscle. An example of such a wild type and mutant protein A is UNC-112 (kindlin) in *C. elegans* muscle, and is shown. Wild type UNC-112 localizes normally to dense bodies (dots) and M-lines (lines), but D382V UNC-112 which does not interact with protein B (in this case, PAT-4), fails to localize (modified from Qadota et al., 2012). As indicated to the right, these transgenic animals are used to assess whether mutant protein A (^*^ and purple) can bind to protein B *in vivo*: protein extracts are prepared from each type of transgenic animal, and immunotagged protein A (wild type, blue, or mutant, purple) is immunoprecipitated (IP) with anti-tag conjugated beads, washed, eluted and then protein B is detected by immunoblot. An example is shown for the UNC-112/PAT-4 interaction in *C. elegans* (modified from Qadota et al., 2012). **(B)** Isolation and characterization of mutations in protein B that restore interaction of protein B with a mutant version of protein A. From top to bottom: a mutant form of protein A (^*^ and purple) does not interact with protein B. Random mutagenesis is used to generate a collection of protein B mutants (indicated by ^#^), and these are tested by yeast 2-hybrid assays for interaction with protein A^*^. One or more such protein B mutants (^#^ and orange) are found to interact with protein A^*^ (denoted by an arrow). Transgenic animals are generated that express mutant protein A^*^ (purple), with either wild type protein B (yellow), or mutant protein B^#^ (orange). Note that protein A and protein B are expressed with different immunotags (e.g., HA or myc, as indicated). These transgenic animals are used in two types of experiments. As indicated below, using an antibody that detects the immunotag for protein A^*^, the ability of mutant protein B^#^ to restore the ability of protein A^*^ to localize in muscle, is assessed by immunostaining. As indicated to the right, these transgenic animals are used to assess whether mutant protein B (^#^ and orange) can restore the ability of protein B to interact with mutant protein A (^*^ and purple) *in vivo*: protein extracts are prepared from each type of transgenic animal, and immunotagged mutant protein A (^*^ and purple) is immunoprecipitated (IP) with anti-tag conjugated beads, washed, and eluted and then protein B is detected by immunoblot.

We then used a heat shock promoter to express in wild type *C. elegans* HA tagged versions of wild type and D382V UNC-112. By western blot using anti-HA, we could detect stable expression of both wild type and D382V UNC-112. By immunofluorescence microscopy using anti-HA, although wild type HA-UNC-112 was properly localized to muscle adhesion sites, D382V HA-UNC-112 was not localized, thus fulfilling our expectation (Figure 3A).

We next reasoned, that if our model that only an open conformation of UNC-112 can bind to PAT-3, then mutations that block the interaction between the N- and C-terminal halves of UNC-112 (constitutively open), might allow D382V UNC-112 to properly localize (Figure 2). Using a similar strategy as described above, random mutations were introduced into UNC-112 N-terminal half, and colonies were screened by yeast 2-hybrid assays for those that interacted with PAT-4, but failed to interact with the UNC-112 C-terminal half. Ninety-three recominated plasmids were obtained, and of these, 87 showed interaction with PAT-4, but 13/87 failed to bind to the UNC-112 C-terminal half. Two plasmids had single residue changes—T346A and E349K (Figure 1). Single and double mutants (with D382V) were generated in full length UNC-112, and tested by 2-hybrid: all bound to PAT-3, and either T346A or E349K bind to PAT-4; only when D382V was added was binding to PAT-4 abolished. The single and double mutant UNC-112 proteins were also ectopically expressed from a heat shock promoter, and the proteins detected with anti-HA by immunofluorescence. All single and double mutants localized to muscle adhesions sites. Therefore, mutations that prevent the N- to C-terminal interaction bypass the requirement of UNC-112 to bind to PAT-4 for proper localization of UNC-112.

It should be pointed out that the isolation of specific non-binding mutants occurs at a high enough frequency to be practical. In our experience, on average, from about 50 mutagenized fragments without premature stops, one or two specific non-binding mutants were isolated. In the cases presented here, we isolated one PAT-4 non-binding UNC-112 mutant from 32 mutants that retained binding to PAT-3, and we recovered 2 C-terminus non-binding mutants of UNC-112 from 87 PAT-4 binding N-terminal mutagenized fragments. A similar ratio has been reported for other types of specific loss-of-function screens in yeast. In the case of yeast conditional lethals (for example, temperature sensitive mutants) screening, for three genes (*RHO1, MSS4, FKS1*), temperature sensitive mutants were isolated at a similar ratio (Homma et al., 1998; Saka et al., 2001; Okada et al., 2010).

## A suppressor mutation approach for finding binding interfaces

### Intermolecular interaction

Because the structure of an UNC-112 (kindlin)/PAT-4 (ILK) has not yet been solved, we decided to take a suppressor mutation approach for revealing an interaction surface for these two proteins. As before, we screened for mutations with the yeast 2-hybrid system, and then tested them in the striated muscle of transgenic nematodes. Beginning with the D382V UNC-112 mutant protein that cannot bind to PAT-4, we sought to isolate “suppressor” mutations in PAT-4 that would allow binding to D382V UNC-112. If successful, we also would have the opportunity to further test our conformational model for the requirement of UNC-112 to bind to PAT-4 in order to have proper localization of UNC-112 to muscle integrin adhesion sites: although D382V UNC-112 cannot localize, a D382V UNC-112 in the presence of a suppressor PAT-4 protein, might localize (Figure 3B).

Using the same general method as described above, we randomly mutagenized full length PAT-4, and screened for PAT-4 mutants than can bind to D382V UNC-112 (Qadota et al., 2014). Upon screening 12,700 colonies, initially 62 were positive for this interaction. Upon re-transformation, 40/62, remained positive. After determining the DNA sequences of 37 such PAT-4 clones, 5 were single amino acid changes, and the others had 2–5 changes each. By comparing these clones with multiple changes, and using *in vitro* mutagenesis to make clones with single residue changes, the total number of clones with missense mutations in single unique residues totaled 9. It is noteworthy that although the entire PAT-4 sequence was mutagenized, all of these mutations reside in the kinase domain, the minimal region required for interaction with UNC-112 (Figure 1).

Fukuda et al. (2009) have reported the crystal structure of human ILK (PAT-4) kinase domain bound to part of α-parvin (PAT-6 in nematodes; Lin et al., 2003). Based on this structure, we developed a homology model of the PAT-4 kinase domain and located the residues mutated in our PAT-4 suppressors. The suppressor mutations cluster in 2 regions on the surface of PAT-4: 6 cluster near the edge of the beta sheet and helix-1 in the N-terminal subdomain, and 3 cluster in helices of the C-terminal subdomain (Qadota et al., 2014). When PAT-4 was substituted for ILK in the ILK/α-parvin complex, α-parvin, and by implication, PAT-6, did not overlap or cover either cluster of PAT-4 suppressor residues. Therefore, either cluster or both could constitute binding surfaces for UNC-112. Moreover, one of the PAT-4 suppressor mutations could permit localization of D382V UNC-112 to integrin adhesion sites in nematode muscle: co-expression of HA-UNC-112 D382V with myc-PAT-4 wild type does not show localization, but HA-UNC-112 D382V with myc-PAT-4 P257L, does show localization (Qadota et al., 2014).

### Intramolecular interaction

Beginning with T346A and E349K UNC-112 (in the N terminal half of UNC-112) that cannot bind to wild type UNC-112 C terminal half, we mutagenized the C terminal half and screened for compensatory mutations that permit T346A UNC-112 to bind to UNC-112 C terminal half. Nine UNC-112 C terminal half mutants containing missense mutations were isolated (Qadota et al. unpub. data). Three of these contained single missense mutations (S644C, N659D, R663Q). In addition, two other mutations (R633Q and I662T) occurred repeatedly in the multiply mutant isolates. When these mutations were tested alone, they were each found to have the suppressor activity. All five residues mutated lie in a 30-residue span (633–663)(Figure 1). All five allow binding to either T346A or E349K UNC-112 N-terminal half proteins. Our interpretation is that residues within the region 633–663 contact residues in 346–349, in the closed conformation of wild type UNC-112. Two of the five residues (R633 and N659) are conserved in human kindlins -1, -2, and -3. Validation of this interpretation awaits determination of the structure of UNC-112, which is currently being pursued.

This type of “positive screening” or “gain-of-function screening” is easier than the previously described “negative screening” (for non-binding mutants). To identify missense mutants that are non-binding, mutagenized fragments without premature stops first need to be recovered. However, for “positive screening,” this step is not required, since a “positive reaction” (i.e., protein interaction) already excludes premature stops or other severe mutations. “Gain-of-function” suppressors are useful for identifying interacting molecules (for a classic example see Adams et al., 1989). In our case, “gain-of-function” suppressors in PAT-4, starting with a non-binding mutant that has a single amino change in UNC-112, was useful for identifying an interacting surface on PAT-4 for two reasons. First, the single amino acid change in the non-binding mutant of UNC-112 is not likely to result in a large conformational change, so that it was more probable that a single amino acid change on the interacting surface of PAT-4 could restore interaction. Second, the random mutagenesis made it possible to identify many such suppressors. Indeed, for the UNC-112-PAT-4 interaction, we identified 9 single amino acid changes, and then after finding that these residues map onto two clusters of a homology model of PAT-4, inferred one or two binding surfaces.

## Broader applications of the method

In the model genetic organisms, such as *S. cerevisiae, C. elegans*, and *Drosophila*, genetic deletions are available and in other systems, knock down by siRNA are available. However, to ask the meaning of protein-protein interactions, deletions or knock downs, resulting in the absence or reduction in level of one protein *in vivo*, is not ideal. One reason is that deletions or knock downs often cause pleiotropic effects. For example, in the case of *unc*-112 and *pat*-4, the null mutants stop development at an embryonic stage, so we cannot examine the effect of these mutants in adult stage muscle, in which structures are more well-defined and amenable to microscopic analysis. Expression of our non-binding mutants in adult stage muscle allows us to examine the function of these protein in the adult stage, but also allows us to examine the essentiality of a protein interaction rather than the essentiality of expression of the protein. Another reason is that some proteins interact with many proteins, so that complete absence of a protein or reduced levels of a protein can result in multiple effects. For example, PAT-4 binds to PAT-6 and UNC-97, not just to UNC-112. Thus, in *pat*-4 null mutants, interactions with PAT-6 or UNC-97 are also abolished, so it is impossible to ask the meaning of the absence of UNC-112 interaction with this type of mutant. In muscle, there are several giant polypeptides (>400,000 Da) consisting of many domains. For example, in *C. elegans* muscle, UNC-89 (the homolog of obscurin in mammals) has been reported to interact with a number of proteins (CPNA-1, MEL-26, LIM-9, and SCPL-1) (Qadota et al., 2008; Xiong et al., 2009; Wilson et al., 2012; Warner et al., 2013). Severe loss-of- function alleles of *unc*-89 also abolish all these interaction, so it would be impossible to determine the meaning of each of these individual protein-protein interactions. One strategy would be to isolate an *unc*-89 mutant that cannot bind to a specific interactor. Reciprocally, we could use methods described here to isolate mutants in an interactor that cannot bind to UNC-89, and after expression in transgenic worms, determine the function of this interaction.

Our strategy for studying protein-protein interactions important for the muscle sarcomere can be generalized, as depicted in Figure 3. This strategy can be utilized in any model system in which transgenics are easily generated and muscle can be easily visualized, such as *Drosophila*, zebrafish, and perhaps *Xenopus*. Indeed, the strategy can be extrapolated to study any protein-protein interaction that occurs in any large cellular structure that can be imaged by light microscopy, for example, synaptic terminals of neurons, or desmosomes of epithelial cells. Here is one example for vertebrate striated muscle: an obscurin isoform, expressed in mouse heart, can be extensively studied in cultured mouse cardiomyocytes. A yeast 2-hybrid screen has revealed that a small segment of this obscurin interacts with protein X. In addition, obscurin and protein X localize to the same regions of the sarcomere (M-lines and Z-disks), and the proper localization of protein X depends on the presence of obscurin (e.g., RNAi mediated knockdown of obscurin, results in mis-loclization of protein X). However, because obscuring is a huge multi-domain protein with many known and expected interactors, this knockdown will also disrupt the localization of many interacting proteins. Random mutatgenesis and yeast 2-hybrid assays are used isolate a missense mutation in protein X (^*^), such that protein X^*^ does not interact with obscurin. Transient transfection is used to express in cardiomyocytes, tagged versions of either wild type or ^*^ mutant protein X, and the tagged versions of these proteins are detected with anti-tag antibodies. Results might show that wild type protein X localizes, but ^*^ mutant protein X does not localize. This strategy demonstrates that interaction of a specific region of obscurin is required for the localization of a particular protein. Such information cannot be obtained by using simple knockdown or even mutations that result in reduced levels or elimination of this obscurin isoform.

## Author contributions

Hiroshi Qadota conceived and developed the method. Hiroshi Qadota and Guy M. Benian obtained data, analyzed it, produced figures, and wrote the manuscript together.

### Conflict of interest statement

The authors declare that the research was conducted in the absence of any commercial or financial relationships that could be construed as a potential conflict of interest.
